# Unannounced Drills Using Patient Actors to Evaluate Health Care Facility Readiness for Infectious Disease Outbreaks — New Jersey, New York, and U.S. Virgin Islands, January–June 2026

**DOI:** 10.15585/mmwr.mm7538a1

**Published:** 2026-10-01

**Authors:** Nang Thu Thu Kyaw, Mary M.K. Foote, Anthony J. Lo Piccolo, Andrew B. Wallach, Christine Y. Chen, Sondra R. Zabar, Jeffrey Wilhite, Lisa Altshuler, Jory Guttsman, John Kulin, Samantha Burke, Laura Hillard, Laura Alves, Tracey Capers, Kathleen Hanley, Colleen C. Gillespie, Vikramjit Mukherjee

**Affiliations:** ^1^Office of Emergency Preparedness and Response, New York City Department of Health and Mental Hygiene, New York, New York; ^2^Career Epidemiology Field Officer Program, Division of State and Local Readiness, Office of Readiness and Response, CDC; ^3^Special Pathogens Program, NYC Health + Hospitals/Bellevue, New York, New York; ^4^Department of Medicine, NYU Grossman School of Medicine, New York, New York; ^5^Public Health Committee, North East Regional Urgent Care Association, Tuckerton, New Jersey.

**Summary**

**What is already known about this topic?**

Unannounced drills using patient actors (mystery patients) have been used to evaluate health care facility management of patients with suspected infectious diseases of public health concern.

**What is added by this report?**

Among 73 such drills conducted in emergency departments, outpatient clinics, and urgent care centers in New Jersey, New York, and the U.S. Virgin Islands using a simulated avian influenza A(H5) scenario, patients were successfully masked and isolated in 44 (60%) drills. The median time from patient arrival to masking was 2 minutes and to isolation was 11 minutes. Exposure-specific screening and use of personal protective equipment were inconsistent.

**What are the implications for public health practice?**

Unannounced drills can identify gaps in health care facility readiness, provide a learning opportunity for staff members, and help guide future public health interventions to improve readiness of regional health care facilities for emerging and reemerging infectious disease threats.

## Abstract

Unannounced drills using professional patient actors (mystery patients) have been used to evaluate health care facility management of patients with infectious diseases of public health concern. Ongoing outbreaks and the expanding role of urgent care centers highlight the need to evaluate the ability of health care facilities to rapidly identify, isolate, and safely manage patients with transmissible diseases. During January–June 2026, 73 drills were conducted in three jurisdictions within U.S. Department of Health and Human Services Region 2 (New Jersey, New York, and the U.S. Virgin Islands) using a simulated avian influenza A(H5) scenario. During these drills, facilities conducted symptom screening in 68 (93%) drills and obtained travel history upon patient arrival in 58 (80%) drills. Among 60 (82%) drills during which the patient was appropriately masked, the median interval from arrival at the facility to masking was 2 minutes (IQR = 1.0–6.0 minutes) (target = 1 minute), and among 52 (71%) drills during which the patient was isolated, the median interval from arrival to isolation was 11 minutes (IQR = 8.0–19.2 minutes) (target = 10 minutes). Both masking and isolation occurred in 44 (60%) drills. The clinical team planned to notify infection prevention and control teams in 40 (54%) drills. The clinician wore all recommended personal protective equipment in 17 (25%) drills. These drills provided an opportunity for facilities to examine implementation of the principles of identification, isolation, and informing. Findings from these drills will help guide future public health interventions to improve readiness among regional health care facilities for emerging and reemerging infectious disease threats.

## Introduction

Epidemics of novel emerging or reemerging infectious diseases have been occurring with increasing frequency in recent decades (*1*). Successive recent outbreaks of viral hemorrhagic fevers, novel coronaviruses (e.g., COVID-19), monkeypox virus disease, measles, and avian influenza A(H5) have reinforced the importance of rapidly recognizing and safely managing patients with potentially severe transmissible infections at health care facility points of entry, to protect health care workers, patients, and surrounding communities (*2*,*3*). The New York City (NYC) Health Department (HD) has employed unannounced drills using professional patient actors (mystery patients) in hospital emergency departments (EDs) and federally qualified health centers to evaluate and strengthen health care preparedness for communicable diseases of public health concern (*4*–*6*). In addition to EDs, urgent care centers (UCCs) have increasingly become a preferred setting for patients seeking care who have acute infections, yet the ability of these facilities to rapidly identify and isolate patients with suspected high-consequence infectious diseases (HCIDs) has not been widely evaluated (*7*).

Preparedness is an ongoing process that requires continuous evaluation and adaptation to remain valid and effective. Therefore, NYCHD partnered with the Regional Emerging Special Pathogen Treatment Center at NYC Health + Hospitals/Bellevue (U.S. Department of Health and Human Services [HHS] Region 2) to conduct unannounced drills in regional hospital EDs, outpatient clinics, and UCCs, using a simulated avian influenza A(H5) scenario to evaluate the health care system’s ability to rapidly and safely identify, isolate, and manage patients with potential HCID without travel-related exposures.

## Methods

### Drill Exercise Design

The New York University (NYU) Standardized Patient Program (*8*) conducted the drills during January–June 2026 using professional patient actors. The exercise design, evaluation guide, and after-action reports were developed in accordance with the U.S. Department of Homeland Security Exercise and Evaluation Program and adapted from previous NYC drills in consultation with an advisory group with expertise in public health, emergency management, infectious diseases, infection prevention, emergency medicine, and primary care (*4*–*6*).

### Drill Objectives

Objectives of the drills were to determine 1) the length of time from patient arrival to identification of a potential HCID through symptom and exposure screening at patient check-in; 2) the length of time from patient arrival to implementation of recommended infection prevention and control (IPC) measures (patient masking and isolation in a room); 3) health care worker adherence to key IPC measures (including hand hygiene and recommended personal protective equipment [PPE] use); and 4) clinician ability to conduct an initial risk assessment and notify the facility IPC team and relevant health departments.

### Invitation to Participate

All hospitals from HHS Region 2 (New Jersey, New York, Puerto Rico, and the U.S. Virgin Islands [USVI]) were invited via newsletters, meetings, and webinars to participate, with the option to conduct drills in their respective EDs or outpatient clinics. UCCs in NYC were recruited through the Northeast Regional Urgent Care Association. The total number of facilities that received invitations was not available.

### Role of the Patient Actor and Assessment Indicators

**Role at the health care facility.** During each drill, a patient actor reported signs, symptoms, and a history compatible with avian influenza A(H5) infection (Box). The patient actor used cosmetics, petroleum jelly, and eye drops to simulate unilateral conjunctivitis. Each drill began when the actor entered the facility and ended after the initial clinical evaluation or the decision to notify the health department. Drills were limited to 2 hours.

BOX **Avian influenza A(H5) scenario used by patient actors in unannounced mystery patient drills — New Jersey, New York, and U.S. Virgin Islands, January–June 2026**

- **Patient actor: **Man or woman; aged 20–28 years
- **Reported signs, symptoms, and history:**
  - Onset previous morning of pink eye (conjunctivitis)
  - Onset overnight or morning of examination:
    - Generalized malaise
    - Fever (101°F [38.3°C]); took Tylenol and Motrin 1 hour before arriving at facility
    - Muscle aches
    - Cough
- **Other relevant history:**
  - Lives with three roommates, none of whom is sick

o No recent travel

- **Patient response if asked about ill contacts at any point during the drill:**
  - “Does a sick bird count?”
  - Response if prompted for the story:
    - Attempted to save a sick duck in Central Park and brought it to the Wild Bird Fund Rehabilitation Center
    - Did not wear gloves
    - Had direct contact with the bird
    - Bird died before patient was able to leave the park
    - Disposed of dead bird in nearest dumpster
  - If patient was not asked about sick contacts at any time during the drill, they shared the sick bird story with the clinician, even if not prompted.

**Data collection.** Patient actors collected data in real time using a predeveloped Research Electronic Data Capture form on mobile devices. During data collection, patient actors maintained the unannounced simulated nature of the exercise.

**Assessment and postdrill activities. **Target times of ≤1 minute from entry to masking and ≤10 minutes from entry to isolation were established to support prompt implementation of CDC-recommended infection control measures at the initial point of entry based on findings from previous measles and Middle East respiratory syndrome drills, in which median times from entry to masking and isolation were 1.5 minutes and 8.5 minutes, respectively. The exercise was considered successful if the patient was masked and isolated in a room regardless of the completion time. Debriefings were conducted with participants at the conclusion of each drill. Postdrill reports were shared with facilities. This activity was reviewed by CDC, deemed not research, and conducted consistent with applicable federal law and CDC policy.*

## Results

Among 73 unannounced drills conducted across 69 EDs, hospital outpatient clinics, and UCCs during January–June 2026, 52 (71.2%) were in NYC, 15 (20.5%) in New York (outside NYC), three (4.1%) in New Jersey, and three (4.1%) in USVI. Puerto Rico did not participate in the drills.

### Symptom Screening, Masking, and Isolation

Results indicate that symptom screening occurred in 68 (93.2%) of the 73 drills, and travel history was obtained in 58 (79.5%) (Table 1). In seven (9.6%) of the drills, participants were asked about exposure history specific to avian influenza A(H5) (Table 2). Symptom screening occurred a median of 4.0 minutes (IQR = 2.0–9.0 minutes) after entry of the patient actor into the facility. Among 60 (82.2%) drills in which patients were masked, masking occurred a median of 2.0 minutes (IQR = 1.0–6.0 minutes) after entry of the patient actor; 25 of 58 (43.1%) patients with time-stamped drills were masked within the 1-minute target time. In 59 (80.8%) of the 73 drills, facilities had masks available in the waiting areas, but availability varied by setting, from 40 of 42 (95.2%) in EDs to 11 of 19 (57.9%) in outpatient sites. Isolation of the patient actor occurred in 52 (71.2%) drills, a median of 11.0 minutes (IQR = 8.0–19.2 minutes) after entry into the facility; nearly one half (25; 48.1%) of drills met the 10-minute target isolation time. Nonclinical staff members conducted a majority of initial screenings: security or reception staff members performed symptom screening in 36 of 68 (52.9%) drills during which symptom screening occurred and provided masks in 38 of 59 (64.4%) drills (Table 2).

**TABLE 1:** Preparedness measures for unannounced mystery patient drills, by facility type — New Jersey, New York, and U.S. Virgin Islands, January–June 2026

Characteristic	No. (total no.), %							
	ED n = 42		Hospital outpatient n = 19		UCC n = 12		Total N = 73	
**Screening at entry**								
Symptoms screened	40 (42)	95.2	17 (19)	89.5	11 (12)	91.7	68 (73)	93.2
Minutes from entry to screening, median (IQR)*	3.0	2.0–7.2	6.0	1.0–16.0	6.5	5.0–10.0	4.0	2.0–9.0
Travel history screened	35 (42)	83.3	14 (19)	73.7	9 (12)	75.0	58 (73)	79.5
Temperature measured	40 (42)	95.2	11 (19)	57.9	11 (12)	91.7	62 (73)	84.9
Masking and signage								
Advised to wear mask	36 (42)	85.7	16 (19)	84.2	8 (12)	66.7	60 (73)	82.2
Minutes from entry to masking, median, IQR*	2.0	0.5–6.0	1.0	1.0–6.5	5.0	2.5–8.0	2.0	1.0–6.0
Masked ≤11 minute^†^	15 (35)	42.9	9 (16)	56.2	1 (7)	14.3	25 (58)	43.1
Masks visibly available at facility	40 (35)	95.2	11 (19)	57.9	8 (12)	66.7	59 (73)	80.8
Signage mandating face coverings visible	28 (35)	66.7	11 (19)	57.9	8 (12)	66.7	47 (73)	64.4
**Isolation**								
Placed in isolation room	24 (42)	57.1	16 (19)	84.2	12 (12)	100.0	52 (73)	71.2
Minutes from entry to isolation, median (IQR)*	9.5	7.8–13.5	20.0	10.0–53.0	9.0	8.5–11.0	11.0	8.0–19.2
Isolated ≤110 minutes^†^	12 (24)	50.0	5 (16)	31.2	8 (12)	66.7	25 (52)	48.1
Placed in airborne infection isolation room (negative pressure)^†^	17 (24)	70.8	6 (16)	37.5	0 (12)	0^§^	23 (52)	44.2
Masked and placed in isolation room	23 (42)	54.8	13 (19)	68.4	8 (12)	66.7	44 (73)	60.3
Clinician encounter								
Seen by NP, PA, or clinician	41 (42)	97.6	15 (19)	78.9	12 (12)	100.0	68 (73)	93.2
Minutes to see a clinician, median (IQR)*	34.0	16.0–57.0	41.0	27.5–63.5	17.0	14.8–18.5	31.0	16.0–53.2
Clinician wore all PPE^¶^	11 (41)	26.8	5 (15)	33.3	1 (12)	8.3	17 (68)	25.0
Clinician did not wear any PPE^†^	2 (41)	4.9	2 (15)	13.3	0 (12)	0	4 (68)	5.9
**Notification and risk identification**								
Notified internal IPC team	21 (42)	50.0	13 (19)	68.4	6 (12)	50.0	40 (73)	54.8
Minutes to IPC notification, median (IQR)*	34.0	20.0–53.0	59.0	25.0–73.5	28.0	25.5–29.5	34.0	21.5–55.0
Notified health department^ †,^**	18 (40)	45.0	11 (19)	57.9	6 (12)	50.0	35 (71)	49.3
Followed internal notification protocols^†^	27 (41)	65.9	15 (18)	83.3	11 (12)	91.7	53 (71)	74.6
Identified patient actor as being at risk for avian influenza^†^	27 (42)	64.3	10 (17)	58.8	8 (12)	66.7	45 (71)	63.4

**Abbreviations:** ED = emergency department; IPC = infection prevention and control; NP = nurse practitioner; PA = physician assistant; PPE = personal protective equipment; UCC = urgent care center.

* Measures are calculated for drills with an applicable prior step completed and a time stamp available. All times are expressed in minutes.

^†^ Denominator was calculated based on an applicable prior step having been completed (e.g., masked ≤1 minute is evaluated among drills where masking was advised); denominators vary slightly across measures because of missing responses. Percentages are calculated based on the number of drills with data available for each measure.

^§^ Urgent care facilities do not maintain negative-pressure capacity, consistent with their role.

^¶^ All PPE was defined as mask, gloves, gown, and eye protection.

** Notification to health department was notional: participants were required to indicate that they would notify the health department, at which point the drill ended. They were not required to actually notify the health department.

**TABLE 2:** Process for unannounced mystery patient drills, by facility type — New Jersey, New York, and U.S. Virgin Islands, January–June 2026

Characteristic	No. (total no.), %							
	ED n = 42		Hospital outpatient n = 19		UCC n = 12		Total N = 73	
**Staff member patient first encountered**								
Security, receptionist, or registrar	28 (42)	66.7	13 (19)	68.4	10 (12)	83.3	**51 (73)**	**69.9**
Greeter	9 (42)	21.4	5 (19)	26.3	0 (12)	—	**14 (73)**	**19.2**
Triage staff member	5 (42)	11.9	1 (19)	5.3	2 (12)	16.7	**8 (73)**	**11.0**
Staff member who performed symptom screening*								
Security, receptionist, or registrar	23 (40)	57.5	9 (17)	52.9	4 (11)	36.4	**36 (68) **	**52.9**
Triage staff member	15 (40)	37.5	5 (17)	29.4	5 (11)	45.5	**25 (68)**	**36.8**
Greeter	1 (40)	2.5	1 (17)	5.9	1 (11)	9.1	**3 (68)**	**4.4**
Patient self–check-in through screen or kiosk	1 (40)	2.5	2 (17)	11.8	1 (11)	9.1	**4 (68)**	**5.9**
Staff member who performed travel history screening*								
Security, receptionist, or registrar	13 (32)	40.6	9 (14)	64.3	0 (9)	—	**22 (55)**	**40.0**
Triage staff members	16 (32)	50.0	3 (14)	21.4	3 (9)	33.3	**22 (55)**	**40.0**
Physician, PA, or NP after triage	1 (32)	3.1	0 (14)	—	1 (9)	11.1	**2 (55)**	**3.6**
Other	1 (32)	3.1	0 (14)	—	4 (9)	44.4	**5 (55)**	**9.1**
Kiosk	1 (32)	3.1	2 (14)	14.3	0 (9)	—	**3 (55)**	**5.5**
**Exposure history screened at triage**								
Contact with contaminated water or surfaces, unpasteurized milk, or animal parts	41 (42)	97.6	19 (19)	100.0	12 (12)	100.0	**72 (73)**	**98.6**
Close contact with ill patient	10 (42)	23.8	4 (19)	21.1	0 (12)	—	**14 (73)**	**19.2**
Contact or prolonged exposure with sick or dead birds, livestock, or other mammals	4 (42)	9.5	3 (19)	15.8	0 (12)	—	**7 (73) **	**9.6**
Staff member who provided mask*								
Security, receptionist, or registrar	21 (36)	58.3	12 (16)	75.0	5 (7)	71.4	**38 (59)**	**64.4**
Triage staff member	5 (36)	13.9	1 (16)	6.2	1 (7)	14.3	**7 (59)**	**11.9**
Greeter	3 (36)	8.3	2 (16)	12.5	1 (7)	14.3	**6 (59)**	**10.2**
Other	1 (36)	2.8	1 (16)	6.2	0 (7)	—	**2 (59)**	**3.4**
Physician, PA, or NP after triage	1 (36)	2.8	0 (16)	—	0 (7)	—	**1 (59)**	**1.7**
**Location patient placed in after triage**								
Private room	21 (42)	50.0	15 (15)	78.9	12 (12)	100.0	**48 (73)**	**65.8**
Curtained cubicle	14 (42)	33.3	0 (15)	—	0 (12)	—	**14 (73)**	**19.2**
Hall	6 (42)	14.3	0 (15)	—	0 (12)	—	**6 (73)**	**8.2**
Other	1 (42)	2.4	4 (15)	21.1	0 (12)	—	**5 (73)**	**6.8**
**Staff member who escorted patient after triage**								
Triage staff member	28 (42)	66.7	14 (19)	73.7	10 (12)	83.3	**52 (73)**	**71.2**
Other	4 (42)	9.5	4 (19)	21.1	1 (12)	8.3	**9 (73)**	**12.3**
Physician, PA, or NP after triage	5 (42)	11.9	1 (19)	5.3	0 (12)	—	**6 (73)**	**8.2**
Security, receptionist, or registrar	5 (42)	11.9	0 (19)	—	0 (12)	—	**5 (73)**	**6.8**
Greeter	0 (42)	—	0 (19)	—	1 (12)	8.3	**1 (73)**	**1.4**
Staff member wore mask during escort	29 (42)	69.0	13 (19)	68.4	10 (12)	83.3	**52 (73)**	**71.2**
Clinician encounter mode*								
Clinician entered the room in person	41 (41)	100.0	13 (15)	86.7	12 (12)	100.0	**66 (68)**	**97.1**
Determined remotely (audio or video)	0 (41)	—	2 (15)	13.3	0 (12)	—	**2 (68)**	**2.9**
Clinician practiced hand hygiene	11 (41)	26.8	5 (15)	13.3	5 (12)	41.7	**21 (68) **	**30.9**
**Type of PPE worn by clinician**								
Mask	34 (41)	82.9	10 (15)	66.7	11 (12)	91.7	**55 (68)**	**80.9**
Eye protection	11 (41)	26.8	6 (15)	40.0	2 (12)	16.7	**19 (68)**	**27.9**
Gloves	34 (41)	82.9	10 (15)	66.7	10 (12)	83.3	**54 (68)**	**79.4**
Gown	15 (41)	36.6	6 (15)	40.0	1 (12)	8.3	**22 (68) **	**32.4**
**Exposure questions asked by clinician**								
Close contact with ill patient	29 (34)	85.3	12 (15)	80.0	8 (12)	66.7	**49 (61) **	**80.3**
Contact or prolonged exposure with sick or dead birds, livestock, or other mammals	21 (34)	61.8	9 (15)	60.0	7 (12)	58.3	**49 (61)**	**60.7**
Contact with contaminated water, unpasteurized milk, or animal parts	5 (34)	14.7	3 (15)	20.0	2 (12)	16.7	**49 (61)**	**16.4**

**Abbreviations:** ED = emergency department; NP = nurse practitioner; PA = physician assistant; PPE = personal protective equipment; RN = registered nurse; UCC = urgent care center.

* Denominator provided reflects drills with an applicable prior step completed (e.g., clinician encounter mode was evaluated among those who met with clinician); denominators vary slightly across measures because of missing responses. Percentages are calculated based on the number of drills with data available for each measure.

### Evaluation of Drills

Overall, 44 (60.3%) drills were deemed successful because both patient masking and isolation were completed. Among the 68 drills with an applicable clinician encounter, clinicians wore all required PPE (e.g., gloves, mask, gown, and eye protection) in 25.0% of drills and did not wear any PPE in 5.9%. The patient actor was identified as being at risk for avian influenza in 45 of 71 (63.4%) drills. Internal IPC staff members were notified or scheduled to be notified in 40 of 73 (54.8%) drills.

## Discussion

Health care facility waiting areas can be high-risk settings for transmission of respiratory viruses if appropriate IPC measures are not implemented (*9*). These unannounced simulation drills provided an opportunity for facilities (i.e., EDs, outpatient clinics, and UCCs in New Jersey, New York, and USVI) to examine real-world applications of the principles of identifying, isolating, and informing^†^ related to HCID screenings, including source control (patient masking), IPC, and notification practices. In addition, the drills facilitated examination of system-level strengths and gaps across ED, outpatient clinic, and UCC settings. The overall median intervals from patient entry to masking (2 minutes) and to isolation (11 minutes) in this study were similar to those in previous drill cycles in NYC; however, fewer than one half of the drills achieved the target times of 1 minute for masking and 10 minutes for isolation. The median time to masking was longest in UCCs (5 minutes), and the median time to isolation was longest in outpatient clinics (20 minutes), underscoring the importance of establishing protocols that incorporate the role of staff members (e.g., the receptionist, registrar, greeter, and security) who would likely be the first to encounter patients to ensure timely implementation of these actions.

This scenario focused on a non–travel-associated HCID, and exposure-specific questions unique to avian influenza were asked considerably less often than were questions about general symptoms or travel history. This finding suggests that screening protocols developed for other respiratory or travel-associated illnesses might not yet be fully adapted to the exposure pathways relevant to domestically spreading or zoonotic pathogens such as avian influenza A(H5). In addition, because some drills were conducted during winter months when seasonal influenza was circulating, clinicians might have been less inclined to consider novel pathogens. These findings highlight the benefit of keeping staff members updated about current infectious disease threats and using standardized protocols and decision-support tools in electronic medical record systems to aid in HCID risk assessments (*10*).

This evaluation also identified inconsistent use of respiratory PPE. Overall, 18% of facilities failed to provide a mask to a visibly coughing patient reporting fever, with UCCs having the lowest compliance; 19% of clinicians did not wear masks or respirators during clinical evaluations, with the lowest compliance in outpatient clinics. Although facilities were encouraged to train staff members, and facility coordinators were provided with a one-page educational handout on avian influenza once the drill was scheduled but before the drill commenced, training was not required, which could affect performance if frontline staff members were not educated about disease recognition and facility protocols. Because the majority of patients encounter security, reception, and registration staff members first (i.e., before seeing a clinical provider), both clinical and nonclinical public-facing staff members who interact with patients could benefit from receiving role-specific, competency-based IPC training.

### Limitations

The findings in this report are subject to at least three limitations. First, not all hospitals in the region participated, and few hospital outpatient and UCC drills were conducted, limiting the generalizability of results. Second, patient actors could not exhibit all signs of avian influenza (e.g., fever and conjunctivitis^§^), which might have limited clinicians' consideration of avian influenza in the differential diagnosis. Finally, participants might have become aware of the drill in advance of or during its performance, potentially compromising the unannounced nature of the drill.

### Implications for Public Health Practice

These drills identified needs for health care workers and health care facility staff member training to achieve earlier identification, masking, and isolation of potentially infectious febrile patients. Inconsistent exposure-specific screening and PPE use occurred in all settings. These findings can guide future public health interventions to improve regional health care readiness for emerging and reemerging infectious disease threats. The drills can also serve as educational and learning opportunities for health care facility staff members. The findings from this report demonstrated the use of this drill format for outpatient settings other than EDs, where patients with acute infections might be examined. Findings identified opportunities to support these outpatient settings in implementing more robust screening, isolation and notification processes, and IPC training. An updated mystery patient drill toolkit is available to help health care facilities and health departments conduct similar drills to test protocols and identify areas for improvement.

## Footnotes

* 45 C.F.R. part 46.102(l)(2),21 C.F.R. part 56; 42 U.S.C. Sect. 241(d); 5 U.S.C. Sect. 552a; 44 U.S.C. Sect. 3501 et seq.

† Identify, isolate and inform principle was originally conceived for frontline health care facilities and providers for Ebola disease and later adapted for management of patients with measles and other diseases of public health concern and promoted by CDC, ASPR, and the National Emerging Special Pathogens Training and Education Center.

§ Although the patient actors tried to simulate conjunctivitis using eye makeup, there is a possibility that it might not have looked like conjunctivitis to the clinician.

## References

[1] Sikkema RS, Koopmans M. Viral emergence and pandemic preparedness in a One Health framework. Nat Rev Microbiol 2026;24:29–44. 10.1038/s41579-025-01243-1 41044181

[2] Brown C, Choi MJ, McLellan S, Shoemaker T. Post-travel evaluation to rule out viral special pathogen infection. In: Halsey ES, Angelo KM, Barnett ED, et al., eds. CDC yellow book, 2026: health information for international travel. Atlanta, GA: US Department of Health and Human Services, CDC; 2025. https://www.ncbi.nlm.nih.gov/books/NBK620879/

[3] Von Stein DL, Barger A, Hennenfent A, Notes from the field: response to a case of travel-associated Lassa fever—Iowa, October–November 2024. MMWR Morb Mortal Wkly Rep 2025;74:194–6. 10.15585/mmwr.mm7411a3 40179054 PMC11970722

[4] Foote MMK, Styles TS, Quinn CL. Assessment of hospital emergency department response to potentially infectious diseases using unannounced mystery patient drills—New York City, 2016. MMWR Morb Mortal Wkly Rep 2017;66:945–9. 10.15585/mmwr.mm6636a2 28910268 PMC5657916

[5] Foote M, Daver R, Quinn C. Using “mystery patient” drills to assess hospital Ebola preparedness in New York City, 2014–2015. Health Secur 2017;15:500–8. 10.1089/hs.2016.0130 28937795

[6] Ali M, Williams MD. No-notice mystery patient drills to assess emergency preparedness for infectious diseases at community health centers in New York City, 2015–2016. J Community Health 2019;44:387–94. 10.1007/s10900-018-00595-5 30604224 PMC6411664

[7] Jacobs-Wingo J, Beatty NL, Jang K, Foote MMK. Assessment of New York City urgent care centers’ emergency preparedness and infection prevention and control practices, 2016–2017. Disaster Med Public Health Prep 2022;16:1059–63. 10.1017/dmp.2021.23 33947497 PMC8568725

[8] Fisher H, Re C, A Wilhite J, A novel method of assessing clinical preparedness for COVID-19 and other disasters. Int J Qual Health Care 2021;33:mzaa116. 10.1093/intqhc/mzaa116 32991675 PMC7543447

[9] Cho SY, Kang JM, Ha YE, MERS-CoV outbreak following a single patient exposure in an emergency room in South Korea: an epidemiological outbreak study. Lancet 2016;388:994–1001. 10.1016/s0140-6736(16)30623-7 27402381 PMC7159268

[10] Lazarus JE, Jerry MS, Germaine L, Don’t know much about geography? decision support for the evaluation of patients with suspected high consequence infectious diseases. Antimicrob Steward Healthc Epidemiol 2025;5:e192. 10.1017/ash.2025.10038 40927242 PMC12415783

